# Preventing Violence toward Sexual and Cultural Diversity: The Role of a Queering Sex Education

**DOI:** 10.3390/ijerph18042199

**Published:** 2021-02-23

**Authors:** Maria Victoria Carrera-Fernández, María Lameiras-Fernández, Nazaret Blanco-Pardo, Yolanda Rodríguez-Castro

**Affiliations:** Faculty of Education and Social Work, University of Vigo, 32004 Ourense, Spain; lameiras@uvigo.es (M.L.-F.); nazblanco@alumnos.uvigo.es (N.B.-P.); yrcastro@uvigo.es (Y.R.-C.)

**Keywords:** sexual and cultural diversity, cis-heteronormativity, ethnocentrism, inclusive LGBTIQ sex education, queer sex education

## Abstract

Background: The prevailing sex education (SE) model falls within a neoliberal prevention- and risk-oriented paradigm. This model ignores the identity dimension of sexuality, is based on the cis-heteronormative and ethnocentric matrix and stigmatizes sexual and cultural diversity; this has significant consequences for sexually and culturally diverse adolescents and youth. In this study, we explored the potential of the identity dimension of SE to prevent violence toward sexual and cultural diversity. Specifically, our objective was to identify the influence of heteronormative and ethnocentric variables on violence exerted against trans* and gender-diverse people and people from minority ethnic groups. Methods: A total of 623 Spanish adolescents with a mean age of 14.73 years and an age range of 13 to 18 years participated in the study. Students completed a questionnaire that included measures regarding violence toward sexual and cultural diversity, gender stereotypes, sexist attitudes and rejection of sexual and cultural diversity. We performed two hierarchical linear regression models. Results: Students who exerted the highest amount of violence toward trans* and gender-diverse people were those who showed the lowest endorsement of expressive traits and the highest endorsement of instrumental traits as well as the highest level of hostile sexist, heteronormative and hostile racist attitudes (the five predictor variables explained 29.1% of the variance of gender-bashing). These same variables—except expressiveness—and benevolent sexism explained 46.1% of the variance of rejection of minority ethnic groups. Conclusions: There is a need for a comprehensive, intercultural, critical and queer SE aimed at transforming the classroom into a space that promotes social transformation through an educational practice that is transgressive and critical of cis-heteronormativity and normative ethnocentrism.

## 1. Introduction

Sex education (SE) in schools plays a key role in the health of adolescents and youth. Hence, when promoting sexual health, it is necessary to consider the different dimensions of sexuality. These include not only the behavioral dimension but also the relational and identity dimensions, which refer to gender identity and expression as well as sexual orientation [1,2,3,4] and pay special attention to social justice and intersectionality [1]. However, the prevailing SE strategies do not fully consider all these dimensions and recommendations, and the most neglected dimension is the identity dimension [5,6]. Next, we explore the current SE model, which is still very far from a truly comprehensive sexual education (CSE), as it is marked by a moralist and highly sanitized approach. In fact, the current preventive model is based on the moral model, which was considered outdated and bygone. The preventive model, however, has only replaced terms such as “sin” and “hell” with others such as “individual responsibility” and “disease”. We analyze how this model has ignored the identity dimension of sexuality, building on the cis-heteronormative and ethnocentric matrix, stigmatizing sexual and cultural diversity and neglecting the very necessary intersectional approach. This has considerable consequences for sexually and culturally diverse adolescents and youth.

### 1.1. From the Conservative to the Neoliberal Model: The Lack of a Truly Comprehensive Sexual Education

There are two main approaches concerning SE models: (1) a conservative or moral model, exemplified by the abstinence-only model in the United States; and (2) a supposedly comprehensive model which, paradoxically, is more akin to a neoliberal, preventive- and risk-oriented model than to a true CSE approach and is not substantially different from the conservative model [6,7]. In the moral or conservative model, abstinence until (heterosexual) marriage is presented as the only legitimate and safe practice to prevent unwanted pregnancies (UWPs) and sexually transmitted infections (STIs); moreover, sexuality is dealt with in the framework of morality, shame, faith, chastity and danger [8]. In the second model, which represents the predominant approach [6,8,9], students are given information on UWPs, STIs and preventive methods, apparently from an objective and “aseptic” perspective based on proven scientific facts and set in a public health context [10]. However, although this model appears to differ from the moral model, its objectives are not substantially different from those of that model, as it continues to focus on the negative aspects of sexuality, shifting the approach from immorality, blame and sin to disease, risks and dangers [10,11]. This model, like the moral model, uses fear as the main learning strategy [6,11], discouraging any sexual activity on the margins of the heteronorm. It considers risk taking and decision making as a responsibility of individuals who choose to make a healthy decision or not, saving or condemning themselves; this disregards the mechanisms of oppression stemming from the intersection of identity categories such as gender, sexual orientation, ethnicity or social class [8,10,12]. Thus, the identity dimension of sexuality has traditionally been ignored in SE [5,6]. In fact, as highlighted by UNESCO [13] in a study on CSE conducted in 48 countries, although 80% of these countries have the strategies and policies required for a true CSE, there is a gap between the recommendations and actual SE practices.

### 1.2. A Cis-Heteronormative and Ethnocentric Sexual Education: The Price of Invisibility and Prejudice

#### 1.2.1. Cis-Heteronormativity and Exclusion of LGBTIQ and Gender-Diverse People

As we just pointed out, gender identity and expression, sexual orientation and the discrimination and violence derived from these categories are usually ignored in SE [5,6]. However, ignoring these issues should not be interpreted to suggest that this SE model is neutral about them. In fact, it is rather the opposite, as it clearly positions itself as a cis-heteronormative model [7,14] and therefore teaches inequality [15], given that education is never neutral and SE is a highly politicized subject [16].

Cis-heteronormativity refers to social norms and discourses on the construction of gender identity and sexual orientation that highlight the natural character of sexual binarism (man/woman) as being congruent with gender binarism (masculine/feminine, respectively) (specifically, the Latin prefix “cis-” means “on the same side” and refers to people whose gender identity or expression aligns with their sex assigned at birth. Conversely, the prefix “trans-”, which means “across from”, refers to people whose gender identity or expression does not align with their sex assigned at birth) and leading to gender identities that are binary, opposed, hierarchical and complementary and therefore necessarily heterosexual [17,18,19]. This cis-heteronormative matrix is also built on sexism and rejection of sexual diversity [20]. In fact, even though cis-heterosexual women reproduce cis-heteronormativity, they are still “the second sex”, as highlighted by Simone de Beauvoir [21]. Along with them, the “other” gender identities and sexual orientations and people who are gender-diverse or perceived as such are also excluded and subjected to violence.

The various studies that have analyzed the relationships between the variables that make up the cis-heteronormative matrix have revealed that (1) adolescents and youth who show a higher endorsement of instrumental gender stereotypes—traditionally associated with masculinity—and a lower endorsement of expressive gender stereotypes—traditionally associated with femininity—are more sexist [22,23], more homophobic [23] and more transphobic [22]; and (2) adolescents and youth with higher scores in sexism are also more homophobic [17,18,19,23,24,25] and more transphobic [22].

Given that SE is mostly focused on the biological dimension and insists on the prevention of UWPs, it is almost exclusively targeted to the sexual relations of cisgender and heterosexual people [26]. It produces discourses in which the sexual desire of women is absent [27] and the virgin/whore binary is transmitted [28,29]. In fact, LGBTIQ-inclusive sex education is not available to most young people [30,31,32,33,34,35], which has significant consequences for the well-being and safety of sexually diverse students at school [23,36,37,38,39,40,41,42].

The situation of trans* (following Platero [43], we use “trans*” as an umbrella term that refers to individuals whose sex assigned at birth does not align with their gender identity, regardless of whether or not they have undergone hormone therapy or sex reassignment surgery), non-binary and gender-diverse (We use the term “gender-diverse” to refer to individuals who express gender nonconformity but do not necessarily ascribe to a trans* identity. We have chosen “gender-diverse” instead of “gender non-conforming”, which has a more negative and less inclusive meaning, following the recommendations of Adams et al. [44]) people is even more negative because their circumstances are ignored to a greater extent than those of sexually diverse people [38,45]; thus, they experience even greater invisibility and situations of oppression and violence at school [46,47].

This SE not only socializes individuals into cis-heteronormativity but also promotes the situations of oppression and violence experienced by LGBTIQ and gender-diverse youth, as shown by studies that have explored the relationship between heteronormativity and violence. Such studies have revealed that (1) the reinforcement of gender stereotypes contributes to gender-related violence in schools [48]; (2) students who show a higher endorsement of instrumental gender stereotypes and a lower endorsement of expressive gender stereotypes exert the most violence toward trans* and gender-diverse people—this violence is also known as gender-bashing [22,23] and bullying [23]; (3) students with higher scores in sexism engage in more gender-bashing and bullying behaviors than others [23]; (4) students who are more homophobic are more engaged in bullying [23,37]; and 5) the most transphobic students engage the most in gender-bashing [22] and bullying [23].

#### 1.2.2. Ethnocentrism and “Sexularism”

Although sexual and gender diversity is absent from SE, the absence of cultural diversity is even greater [49]. Even in countries such as Sweden or Canada, where SE is more inclusive regarding sexual diversity than in other countries, cultural diversity is still neglected. In fact, notions of cultural difference are ignored or converted into superficial complements of gender and sexual diversity at best [49,50,51]. However, as reported by Mukoro [52], when you address sexuality in education, you inevitably deploy a specific sexual culture. Thus, although SE is strongly cis-heteronormative, it is no less ethnocentric. Ethnocentrism refers to social norms and discourses on race and ethnicity and more broadly to culture. This implies a hierarchization of groups and cultures, taking one’s own cultural group as a reference and as being superior [53], and is closely associated with racism/xenophobia [54].

In this ethnocentric context, Scott [55] argues that sexularism is a strategy proper to SE that presents values regarding sexuality that have a secular and Western character as being good, progressive and committed to social justice; by contrast, values associated with other religions and cultures are presented as inadequate and dangerous [10,56]. Specifically, equality and tolerance are used as ethnic markers of being Western, whereas patriarchy, violence and homophobia are associated with the culture of others [57]. These ethnocentric discourses convey homogeneous and monolithic perceptions of young Black males as sexual offenders [58,59] and racialized young men and women as sexually precocious [60], oversexed and over-reproductive [61], and more generally as being problematic and at risk. Overall, race—understood as any race that is not White and Western—is often presented as a risk factor for UWPs and STIs, ignoring the structural factors that explain the experiences of these youth [2,51].

Moreover, this SE does not only socialize individuals into ethnocentrism but also promotes violence. Many studies have underlined the relationship between ethnocentrism and the racism/xenophobia it leads to and violence, including bullying [22,23,25] and hate crimes [62]. The intersectional approach, also ignored in SE, is also particularly important [63,64]. It argues that identity is made up of a constellation of categories and underlines that oppression cannot be analyzed considering only one category of identity or the sum of different categories of identity; instead, this requires considering their intersection [65]. Thus, SE fails to include Black LGBTIQ youth and adolescents, who feel underrepresented, unsupported, stigmatized and bullied in experiences of SE at school [66]. This reproduces gender, sexual and racial hierarchies, which proves that cis-heteronormativity and ethnocentrism operate jointly in SE [61,67]. In fact, strong relationships have been identified between the cis-heteronormative matrix and ethnocentrism. Specifically, it has been observed that the most racist adolescents and youth show the highest endorsement of masculine gender stereotypes [22,23] and express the most sexist [22,23,24,25,68,69], homophobic [24,25,69] and transphobic attitudes [22]. It has also been observed that the most racist youth exert the most violence toward trans* and gender-diverse people [22,23] and bullying [23].

### 1.3. The Current Study

The objective of this study was to analyze the potential of a truly comprehensive and inclusive SE—that is, an SE that pays special attention to the identity dimension of sexuality, considering gender, sexual orientation and race/ethnicity—to prevent violence toward sexual and cultural diversity. Specifically, we intended to assess the joint influence of cis-heteronormative variables, such as gender stereotypes (expressiveness and instrumentality), sexist attitudes (hostile and benevolent) and attitudes toward trans* and gender-diverse people, and ethnocentric variables (classical or hostile racism) on the rejection and violence exerted against sexual and cultural diversity. We are not aware of any studies to date that have analyzed the influence of these variables on violence toward sexual and cultural diversity in adolescents jointly and in the framework of CSE. Therefore, this study contributes innovative ideas that may be especially relevant to understanding violence toward trans* and gender-diverse people and people from minority ethnic groups and to designing truly comprehensive SE policies and strategies that can effectively prevent such violence.

Based on the above-mentioned premises, we expected adolescents who exerted more violence toward sexual diversity and showed a higher rejection of minority ethnic groups to describe themselves as being less expressive and more instrumental and exhibit more hostile sexist and racist attitudes and more negative attitudes toward trans* and gender-diverse people. In short, we expected a true CSE to be a key strategy to prevent cis-heteronormative and ethnocentric violence.

## 2. Materials and Methods

### 2.1. Participants

The sample was composed of 623 adolescents from northwestern Spain—53% girls and 47% boys—who were attending the last two years of compulsory secondary education at seven schools in urban areas. Five of these schools were public and two were subsidized private schools. Mean age was 14.73 years (SD: 0.90) and age ranged from 13 to 18 years. As regards cultural/ethnic identity, 82.7% considered themselves Spanish, 77.2% Galician, 5.9% South American, 0.8% African, 0.3% Asian, 0.3% Roma and 7.9% identified with other ethnic-cultural categories.

### 2.2. Instruments

The students completed a self-reported questionnaire that included measures regarding violence toward sexual and cultural diversity, gender stereotypes, sexist attitudes and rejection of sexual and cultural diversity.

As regards dependent variables, we included the following scales:

*Gender-bashing subscale* (part of the Genderism and Transphobia Scale [70], short version by Carrera et al. [5]). It measures violent behaviors toward trans* and gender-diverse people (e.g., “I have behaved violently toward a woman because she was too masculine”). It consists of 6 items that are responded to on a Likert scale from 1 (strongly disagree) to 7 (strongly agree). Higher scores indicate greater engagement in violent behaviors.

*Rejection of Minority Ethnic Groups scale* (Based on the Escala de Intolerancia y Justificación de la Violencia hacia Minorías [Scale of intolerance and justification of violence toward minorities]. This scale is part of the Cuestionario de Actitudes hacia la Diversidad y la Violencia [Questionnaire on attitudes toward diversity and violence] [71]). It is composed of 8 items that measure rejection of minority ethnic groups, specifically Roma or Gypsy people (antigypsyism), Chinese people (sinophobia) and Muslim people (islamophobia) (e.g., “It would be good to build specific housing areas for Gypsy people where they can live the way they want, thus avoiding conflicts with the rest of society”, “Chinese people get organized among themselves to gain power and take it from others” or “Muslims are sexist”). Responses are provided on a Likert scale from 1 (strongly disagree) to 5 (strongly agree). Higher scores indicate more rejection of minority ethnic groups.

As regards independent variables, we included the following scales:

*Femininity Trait Index* [72]. This scale uses 10 items to analyze expressive personality traits or feminine gender stereotypes used to describe students (e.g., “sensitive to others’ needs”). It is responded to on a Likert scale from 1 (never describes me) to 7 (always describes me). Higher scores reflect a higher endorsement of feminine gender stereotypes.

*Masculinity Trait Index* [72]. This scale uses 10 items to analyze instrumental personality traits or masculine gender stereotypes used to describe students (e.g., “dominant”). It is answered on a Likert scale from 1 (never describes me) to 7 (always describes me). Higher scores indicate a higher endorsement of masculine gender stereotypes.

*Ambivalent Sexism Inventory* [73], short version by Rodríguez, Lameiras and Carrera [74]. It is composed of the Hostile Sexism subscale, which measures sexist attitudes toward women with a negative affective tone (e.g., “Women exaggerate problems they have at work”), and the Benevolent Sexism subscale, which measures paternalistic sexist attitudes. These attitudes are characterized by a benevolent and positive tone that reinforces women who embrace traditional gender roles but continues to relegate them to a different and lower level (e.g., “Many women have a quality of purity that few men possess”). Each subscale includes 6 items that are responded to on a Likert scale from 1 (strongly disagree) to 6 (strongly agree). Higher scores indicate a higher level of hostile and benevolent sexist attitudes.

*Genderism/Transphobia subscale* (part of the Genderism and Transphobia Scale [70], short version by Carrera et al. [5]). It includes 6 items that analyze beliefs and attitudes regarding trans* and gender-diverse people (e.g., “Feminine boys make me feel uncomfortable”). Students respond on a Likert scale from 1 (strongly disagree) to 7 (strongly agree). Higher scores indicate more negative attitudes toward sexual diversity.

*Classical/Hostile Racial Prejudice subscale* (included in the Classical and Modern Racial Prejudice Scale, by Akrami, Ekehammar and Araya [75]). It is composed of 8 items that measure classical or hostile racism, that is, blatant or old-fashioned racism expressed directly and openly (e.g., “Immigrants do not take care of their personal hygiene”). Responses are provided on a Likert scale from 1 (strongly disagree) to 5 (strongly agree) Higher scores indicate more hostile racist attitudes.

Table 1 shows the descriptive statistics and the reliability of the scales in this study.

### 2.3. Procedure

We used a correlational design with a random sampling cross-sectional survey. We randomly selected seven compulsory secondary education schools out of a total of 21 schools in a town in northwestern Spain. From each school, we selected full groups of students in the same class attending either of the last two years of compulsory secondary education. The questionnaire was individual, anonymous, voluntary and self-administered. Students completed it under the supervision of trained staff during school class hours. Prior to conducting the survey, the parents or legal guardians of students gave written prior informed consent. After an initial explanation of the format of the questionnaire and how to answer it, students answered it and questions were solved by asking the researchers individually. The mean time it took to answer the questionnaire was 40 min. The study complied with the Declaration of Helsinki [76] and its subsequent updates on ethical standards for research with humans regarding the following issues: data collection, including prior informed consent, the right to information, the protection of personal data, the guarantee of confidentiality, non-discrimination and no compensation for participation.

### 2.4. Data Analyses

First, to analyze the relationships between the different variables of the study, we calculated Pearson’s correlation coefficients (*r*). Next, we performed two hierarchical linear regression analyses to predict violence toward trans* and gender-diverse people (gender-bashing) as well as rejection of minority ethnic groups based on the different variables analyzed. Specifically, in both regression models, we included gender stereotypes—expressiveness and instrumentality—in Step 1; we included sexist attitudes—both hostile and benevolent—in Step 2; we added attitudes toward trans* and gender-diverse people in Step 3; and we included hostile racism in Step 4. In each step, the proportion of unique effect that each predicted variable contributed was shown by beta coefficients (β) and Student’s t-test. We used the coefficient of determination (*R*^2^), adjusted coefficient (Δ*R*^2^), ANOVA (*F*) and *p*-values to explore significant effects in the regression model.

## 3. Results

### 3.1. Relationships between Variables

As shown in Table 2, all correlations between the study variables, on one side, and gender-bashing and rejection of ethnic minority groups, on the other, were significant. In addition, gender-bashing showed a strong positive correlation with genderism; a moderate positive correlation with hostile and benevolent sexism and hostile racism; a weak negative correlation with expressiveness; and a weak positive correlation with instrumentality. Rejection of ethnic minority groups exhibited a strong positive correlation with hostile sexism, genderism/transphobia and hostile racism; a moderate positive correlation with benevolent sexism; a weak negative correlation with expressiveness; and a weak positive correlation with instrumentality. In addition, we found strong positive correlations between hostile sexism and benevolent sexism, hostile sexism and genderism/transphobia, benevolent sexism and genderism/transphobia, and genderism/transphobia and hostile racism. Interestingly, we also found moderate positive relationships between the two dependent variables—gender-bashing and rejection of ethnic minority groups—and between hostile sexism and hostile racism, and benevolent sexism and hostile racism. Expressiveness and hostile racism behaved the same way but exhibited a negative relationship.

### 3.2. Hirarchical Linear Regression Model

We performed two hierarchical linear regression analyses to predict gender-bashing and rejection of minority ethnic groups. As mentioned above, in both, we included gender stereotypes—expressiveness and instrumentality—in Step 1; we included sexist attitudes—both hostile and benevolent—in Step 2; we added attitudes toward trans* and gender-diverse people in Step 3; and we included hostile racism in Step 4.

As regards gender-bashing (see Table 3), in Step 1, results confirm that expressiveness and instrumentality were significant predictors. Specifically, male and female adolescents who exercised the most violence toward trans* or gender-diverse people described themselves as being significantly less expressive (*β* = −0.19; *p* < 0.001) and more instrumental (*β* = 0.17; *p* < 0.001); gender stereotypes explained 6.1% of the variance. Step 2, in which we included hostile and benevolent sexist attitudes, increased the fit of the model by 12.4%; specifically, students who expressed significantly more hostile sexist attitudes (*β* = 0.27; *p* < 0.001) and more benevolent sexist attitudes (*β* = 0.13; *p* < 0.01) were the most violent toward trans* or gender-diverse people. In Step 3, we included genderism/transphobia, which significantly increased the fit of the model (9.6%); in fact, students with more negative attitudes toward trans* and gender-diverse people (*β* = 0.38; *p* < 0.001) engaged the most in situations of violence toward this group. Finally, in Step 4, we included negative attitudes toward cultural diversity (i.e., hostile racism), which weakly but significantly increased the fit of the model (1%); in fact, students who expressed more hostile racist attitudes (*β* = 0.11; *p* < 0.01) exerted more violence toward trans* or gender-diverse people. Taken together, all the variables included in the model except for benevolent sexism explained 29.1% of the variance in gender-bashing.

As regards rejection of minority ethnic groups (see Table 4), in Step 1, results show that expressiveness and instrumentality were significant predictors. Specifically, students who reported a greater rejection of minority ethnic groups showed a lower endorsement of expressive gender stereotypes (β = −0.14; *p* < 0.001) and a higher endorsement of instrumental gender stereotypes (β = 0.18; *p* < 0.001); gender stereotypes explained 5.2% of the variance. In Step 2, in which we included hostile and benevolent sexist attitudes, we observed that students who expressed significantly more sexist attitudes—both hostile (β = 0.29; *p* < 0.001) and benevolent (β = 0.20; *p* < 0.001)—showed the highest rejection of minority ethnic groups. In this step, the fit of the model increased by 17.7%. In Step 3, we found that genderism/transphobia (β = 0.27; *p* < 0.001) was a significant predictor of rejection of minority ethnic groups; specifically, male and female adolescents who expressed more negative attitudes toward trans* or gender-diverse people showed the greatest rejection of Gypsy (Roma), Chinese and Muslim people. In this step, the fit of the model increased by 4.8%. Finally, in Step 4, we included classical racism, that is, hostile racism (β = 0.49; *p* < 0.001), and found that students who exhibited more hostile or traditional racist attitudes showed the highest rejection of minority ethnic groups; this step increased the fit of the model by 18.4%. Taken together, all the variables included in the model, except expressiveness, accounted for 46.1% of the variance of rejection of minority ethnic groups.

## 4. Discussion

In this study, we analyzed the potential of the identity dimension of SE to prevent violence toward sexual and cultural diversity. Specifically, our objective was to identify the influence of cis-heteronormative and ethnocentric variables on violence toward trans* and gender-diverse people and people from minority ethnic groups. To do so, we conducted two hierarchical linear regression analyses. In both cases, gender stereotypes, namely, expressiveness and instrumentality, were included in Step 1; sexist attitudes, both hostile and benevolent, were included in Step 2; attitudes toward trans* and gender-diverse people (genderism/transphobia) were included in Step 3; and, finally, hostile racism was included in Step 4.

Results show that students who exerted the most violence toward trans* and gender-diverse people were those who showed a lower endorsement of feminine gender stereotypes and a higher endorsement of masculine gender stereotypes; these participants also expressed more hostile sexist attitudes and more negative attitudes toward sexual and cultural diversity. These variables explained 29.1% of the variance of gender-bashing. Benevolent sexism along with the above-mentioned variables, except expressiveness, also explained rejection of minority ethnic groups (42.1% of the variance).

In line with the premises and studies mentioned above, instrumental gender stereotypes, traditionally associated with hegemonic masculinity, were closely related to violence [22,23,37,48]. They specifically predicted violence toward trans* and gender-diverse people [22] as well as rejection of minority ethnic groups [22,23], including antigypsyism, sinophobia and islamophobia. Conversely, expressiveness, traditionally associated with feminine stereotypes, was a protective factor that correlated with greater acceptance of others; in fact, students who showed a lower endorsement of feminine gender stereotypes were the most violent toward sexual diversity [22]. This is consistent with studies that have explored the influence of expressiveness on participation as a perpetrator in situations of school bullying [23,37].

Along with masculine gender stereotypes, hostile sexism predicted violent behaviors toward trans* and gender-diverse people and rejection of minority ethnic groups. These results are consistent with those of previous studies that have found unequivocal links between hostile sexism and bullying at school [23,37], hostile sexism and gender-bashing [22,70], and hostile sexism and rejection of minority ethnic groups [22,23,24,25,68,69]. Benevolent sexism also predicted rejection of cultural diversity but not violence toward sexual diversity. Along these lines, benevolent sexism showed an ambivalent behavior in the prediction of violence, acting sometimes as a risk factor [77] and other times as a protective factor [22,37]. In this regard, the positive and paternalistic affective tone of benevolent sexism may explain its supposedly protective character. However, this should be taken with caution, as we observed in this study, because despite its apparent affective nature, it relegates women to a place that is not only different but also inferior.

To continue with the cis-heteronormative variables, negative attitudes toward trans* and gender-diverse people (genderism/transphobia) predicted violence toward sexual diversity [22,70] and rejection of cultural diversity [78]. Moreover, hostile racism predicted not only rejection of cultural diversity [62] but also violence toward trans* and gender-diverse people [22,23,78]. These findings highlight the overlapping that exists between the cis-heteronormative matrix and ethnocentrism [61,67] and therefore the intersectional nature of oppression [63,64].

These results are relevant not only to better understand and prevent the violence experienced by sexually and culturally diverse people but also to especially guide the development of future SE practices. They show that there is an urgent need for a truly comprehensive, intercultural, critical and queer SE [79] that addresses identity-related variables, is aimed at reducing gender stereotypes and promotes expressive values for all students as well as egalitarian attitudes and positive attitudes toward sexual and cultural diversity.

This SE transcends the binarism of the two classical models—the conservative model and the neoliberal model—and positions itself at the intersection of a critical and post-modern paradigm [80]. This paradigm is critical along the lines of the pedagogy of Paulo Freire [81], assuming that education is essentially aimed at humanizing students and that its ultimate goal is utopia, that is, the transformation of reality. It is also a post-modern paradigm, as it criticizes modern school for being at the service of the apparatuses of power, condemning the fact the curriculum is not organized to eliminate inequalities but rather to legitimize and regulate them, and ignoring differences of class, ethnic group, gender or sexual orientation or subordinating them to the imperatives of a linear history and culture [82].

It is, in short, a queer SE that emerges in the philosophical context of queer theory [18,19]. Its main premise is not to promote tolerance of minority groups but to contribute to problematizing education. In other words, this refers to conducting a critical analysis of the construction of normality and of how oppression operates through the repetition of norms on the intelligibility of identity and also through the exclusion of people who exceed the limits of such intelligibility [80]. Thus, this queer SE stems from questioning the teaching practices of standardization, paying attention to the exclusions that they generate when they deny or alienate the self-determination of people and peoples to name their identity [83] and position cis-heteronormativity, whiteness and Western culture as the center and norm of all things [84]. It is oriented toward the construction of other alternative spaces of identification and experience of desire [85] as well as the exchange of cultural languages and values based on an intercultural model [84,86]. This queer SE has two objectives: (1) ending the imposition of a single, fixed and coherent identity which, as highlighted by Butler [18], is the breeding ground of violence and the exclusion of identities perceived as unintelligible, abject or threatening for the subject’s coherence; and (2) “thinking the world” beyond the rigid binomial: man-masculine-heterosexual/woman-feminine-heterosexual; or White/Black, non-Gypsy/Gypsy, Western/underdeveloped or native/immigrant. Both objectives could be summarized into the need to shift from the status quo to the “status queer” [87].

As regards the main strategies that should be used in this type of SE experience, it is worth underlining all those that imply the inclusion of LGBTIQ realities and cultural diversity, specifically, (1) including identity topics as cross-cutting in the curriculum; more holistic discussions on sexuality; and more information on the development and construction of identity [88]; (2) not assuming the idea that there are two sexes and two sexes only [80,87]; (3) decoupling sexual anatomy or assigned sex from gender identity; (4) not assuming the gender identity or sexual orientation of the students based on their appearance; (5) not assuming the cis-heterosexuality of all students, acknowledging that there are people who are not cis-heteronormative in the classroom; (6) taking care of language and using inclusive or gender-neutral language [89]; (7) including examples that recognize a broad variety of personal identities and relational and family models, both regarding gender identity and sexual orientation and racial/ethnic diversity [2,89]; (8) not ignoring the sociocultural variables that explain oppression, assuming that sex, gender identity, sexual orientation or race/ethnicity are not the risk factors [2,51]; (9) acknowledging the intersectional nature of oppressions [10,61,65], creating safe spaces and training teachers that unconditionally support their students [88,90].

The benefits of this type of inclusive SE program are broad and indisputable: the reduction in homophobic and gender-related bullying [9,91] as well as hostile school environments [9,26]. More specifically, SE programs that strive to increase the acceptance of trans* and gender-diverse people reduce the violence experienced by these youth [92]. These programs have also been found to contribute to the reduction in school absenteeism and mental health problems, including suicidal thoughts and suicide attempts, and to the increase in healthy behaviors, including a reduction in drug and alcohol use and the number of sexual partners and an increase in the prevention of UWPs and STIs [9].

### Limitations and Future Research Directions

Despite the contributions of the study, there are also several limitations that can guide future research. The cross-sectional and correlational nature of this study limits the conclusions of causality that can be drawn between variables. Therefore, in future studies, such causal relationships should be confirmed with longitudinal and experimental studies. Moreover, data were obtained through self-reported scales administered to adolescents and youth at schools; in future studies, they could be complemented by the contributions of teachers and families and also with the triangulation of other data collection methods, preferably qualitative ones. Furthermore, it would be necessary to conduct rigorous assessments of SE programs that are truly comprehensive and implemented systematically—rather than occasionally—with quasi-experimental designs including quasi-control groups. Ideally, they should be multi-method assessments including quantitative and qualitative methodologies; they should give a voice to the students involved and explore their opinions and experiences about the program in detail. Finally, although this study had an intersectional approach, it is obvious that intersectionality encompasses more than gender identity, sexual orientation and race/ethnicity. Therefore, this analysis will need to be completed by the influence of other identity categories such as social class or disability to guide SE policies and strategies that are truly inclusive and committed to social justice and human rights.

## 5. Conclusions

In this study, we analyzed the potential of the identity dimension of SE to prevent violence toward sexual and cultural diversity. Specifically, our objective was to identify the influence of cis-heteronormative and ethnocentric variables on violence exerted toward trans* and gender-diverse people and people from minority ethnic groups. In short, we intended to analyze the potential of a true CSE that extends beyond a neoliberal and prevention- and risk-oriented SE model. The results derived from the two hierarchical linear regression models confirm that students who exerted the most violence toward trans* and gender-diverse people and showed the highest rejection of minority ethnic groups were those who described themselves more significantly based on gender stereotypes related to hegemonic masculinity and who showed more hostile sexist attitudes and more negative attitudes toward sexual and cultural diversity. Benevolent sexism also explained rejection of minority ethnic groups.

Our study is consistent with studies that have highlighted the intersectional nature of oppressions. It proves the need to conduct an SE contextualized within a comprehensive model that considers variables related to identity, aiming at reducing gender stereotypes, promoting positive attitudes toward equality, making sexual and cultural diversity visible and taking it into account. In short, we are referring to an intercultural, critical and queer SE whose objectives and contents are not aimed at ensuring that hegemonic groups (“the center”) tolerate the others (“the margins”) but rather at transforming the classroom into a space that promotes social change through a transgressive educational practice that is critical of the authoritarian structures of the school, questions cis-heteronormativity and normative ethnocentrism and seriously challenges normative identity categories, valuing other ways of being and positioning oneself as a human being. This will help to prevent violence toward people who transgress the gender norm and violence against cultural diversity. Therefore, it will contribute to the development of more just, democratic and egalitarian schools.

## Figures and Tables

**Table 1:** Descriptive statistics and reliability of the scales.

	*Mean*	*SD*	*α*
Gender-bashing	1.34	0.69	0.85
Rejection of minority ethnic groups	2.35	0.82	0.85
Expressiveness	5.53	0.83	0.86
Instrumentality	4.51	1.1	0.86
Hostile sexism	2.50	0.99	0.75
Benevolent sexism	2.99	1.0	0.77
Genderism/Transphobia	2.45	1.3	0.84
Hostile racism	2.07	0.55	0.71

**Table 2:** Correlations between the study variables.

	1	2	3	4	5	6	7
(1) Gender-bashing							
(2) Rejection of minority ethnic groups	0.33 **						
(3) Expressiveness	−0.180 **	−0.133 **					
(4) Instrumentality	0.161 **	0.178 **	0.045				
(5) Hostile sexism	0.382 **	0.426 **	−0.168 **	0.166 **			
(6) Benevolent sexism	0.274 **	0.351 **	−0.023	0.092 *	0.481 **		
(7) Genderism/Transphobia	0.493 **	0.455 **	−0.179 **	0.175 **	0.480 **	0.502 **	
(8) Hostile racism	0.346 **	0.616 **	−0.257 **	0.103 *	0.338 **	0.237 **	0.451 **

*Note:* Gender-bashing = higher scores indicate more abusive behaviors toward trans* and gender-diverse people; rejection of minority ethnic groups = higher scores indicate more rejection of Gypsy people, Chinese people and Muslim people; expressiveness = higher scores indicate a higher endorsement of feminine gender stereotypes; instrumentality = higher scores indicate a higher endorsement of masculine gender stereotypes; hostile sexism = higher scores indicate more hostile sexist attitudes; benevolent sexism = higher scores indicate more benevolent sexist attitudes; genderism/transphobia = higher scores indicate more transphobia; hostile racism = higher scores indicate higher hostile racist attitudes. * *p* < 0.05; ** *p* < 0.01.

**Table 3:** Hierarchical linear regression analysis predicting gender-bashing.

	Step 1		Step 2		Step 3		Step 4	
Predictor	β	t	β	t	β	t	β	t
Expressiveness	−0.19	−4.81 ***	−0.14	−3.81 ***	−0.84	−2.37 *	−0.06	−1.80 +
Instrumentality	0.17	4.34 ***	0.11	2.95 **	0.07	1.96 *	0.06	1.88 +
Hostile sexism			0.27	6.41 ***	0.17	4.19 ***	0.16	3.80 ***
Benevolent sexism			0.13	3.23 **	−0.01	−0.20	−0.01	−0.19
Genderism/Transphobia					0.39	9.08 ***	0.35	7.77 ***
Hostile racism							0.11	2.93 **
*F (df, df error)*	20.11 (2, 620) ***		35.02 (4, 618) ***		48.21 (5, 617) ***		42.10 (6, 616) ***	
*R* ^2^	0.061 ***		0.185 ***		0.281 ***		0.291 ***	
Δ*R*^2^			0.124 ***		0.096 ***		0.010 **	

*Note:* + *p* < 0.010; * *p* < 0.05; ** *p* < 0.01; *** *p* < 0.001.

**Table 4:** Hierarchical linear regression analysis predicting rejection of minority ethnic groups.

	Step 1		Step 2		Step 3		Step 4	
Predictor	β	t	β	t	β	t	β	t
Expressiveness	−0.14	−3.67 ***	−0.10	−2.58 *	−0.05	−1.48	0.03	1.02
Instrumentality	0.18	4.71 ***	0.11	3.19 **	0.08	2.46 *	0.07	2.41 *
Hostile sexism			0.29	7.07 ***	0.22	5.35 ***	0.16	4.27 ***
Benevolent sexism			0.20	4.96 ***	0.10	2.35 *	0.10	2.79 **
Genderism/Transphobia					0.27	6.40 ***	0.10	2.53 *
Hostile racism							0.49	14.48 ***
*F (df, df error)*	16.90 (2, 620) ***		45.94 (4, 618) ***		47.32 (5, 617) ***		87.71 (6, 616) ***	
*R* ^2^	0.052 ***		0.229 ***		0.277 ***		0.461 ***	
Δ*R*^2^			0.177 ***		0.048 ***		0.184 ***	

Note: * *p* < 0.05; ** *p* < 0.01; *** *p* < 0.001.

## Data Availability

The data presented in this study are available from the corresponding author on reasonable request.
